# Genome-Wide Identification and Expression Profiling of the *ARF* Gene Family During Seed Germination in Sesame (*Sesamum indicum* L.) Under Abiotic Stresses

**DOI:** 10.3390/ijms27125470

**Published:** 2026-06-17

**Authors:** Yanxin Deng, Junchao Liang, Pan Zeng, Zhiqi Wang, Xiaowen Yan, Wenliang Wei, Jian Sun

**Affiliations:** 1 MARA Key Laboratory of Sustainable Crop Production in the Middle Reaches of the Yangtze River, Engineering Research Center of Ecology and Agricultural Use of Wetland, College of Agriculture, Yangtze University, Ministry of Education, Jingzhou 434025, China; dengyanxin0717.stu@yangtzeu.edu.cn; 2 Jiangxi Province Key Laboratory of Oilcrops Genetic Improvement, Crops Research Institute of Jiangxi Academy of Agricultural Sciences, Nanchang Branch of National Center of Oilcrops Improvement, Nanchang 330200, China; njljc@163.com (J.L.); 15271750715@163.com (P.Z.); 18679145645@163.com (Z.W.); yanxiaowen1983@126.com (X.Y.)

**Keywords:** auxin signaling, *Sesamum indicum* L., seed germination, abiotic stress

## Abstract

Auxin response factors (ARFs) are pivotal regulators mediating plant growth, development, and abiotic stress responses, especially during seed germination under stressful conditions. However, the ARF gene family has not been thoroughly studied or characterized in sesame. The identification and characterization of ARF family members in the sesame genome were analyzed by bioinformatics methods, and the expression patterns of sesame *ARF* genes were assessed by quantitative real-time PCR. In this study, a total of 23 *ARF* genes were identified in the sesame genome, distributed unevenly across 12 chromosomes. Additionally, 15 segmental duplication events were detected. Phylogenetic analysis classified the *SiARF* genes into four subfamilies, with members within each subgroup sharing conserved structural features and motif compositions. Promoter analysis revealed multiple cis-acting elements associated with plant growth, phytohormone responses, and stress responses. Expression profiling demonstrated distinct tissue-specific expression patterns among the *SiARF* genes. Notably, *SiARF5* and *SiARF15* showed predominant expression in seeds 5 days after pollination, whereas *SiARF14* exhibited broad expression in roots, stems, leaves, and seeds germinated for 24 h. QRT-PCR analysis identified eight *SiARF* genes exhibiting biphasic expression patterns during seed germination under abiotic stresses, characterized by initial downregulation and subsequent upregulation. Among them, *SiARF11* showed significant induction under all three stress conditions, while *SiARF9* was specifically upregulated under salt stress, suggesting their critical roles in stress response regulation. These findings provide a foundation for further investigation into Auxin-mediated responses to abiotic stress during seed germination in sesame.

## 1. Introduction

High temperature, drought, and salinity are major abiotic stresses in agricultural production, significantly impairing crop growth, development, yield, and quality [1]. Seed germination, a critical initial phase of the crop life cycle, is highly susceptible to inhibition by these abiotic factors [2]. Phytohormones play a central role in regulating seed germination, and the inhibitory effects of high temperature, drought, and salinity are primarily mediated through alterations in the dynamic balance among multiple phytohormones, including abscisic acid (ABA), gibberellins (GA), auxin, ethylene, cytokinins, brassinosteroids (BR), salicylic acid, and jasmonic acid [3].

Auxin plays multiple critical roles in regulating plant growth and development. Auxin signaling is primarily controlled by two gene families: Auxin response factors (ARFs) and Aux/indole-3-acetic acid (Aux/IAAs) [4]. *ARFs* are essential transcription factors in the auxin signaling pathway, regulating diverse developmental processes, such as seed germination, embryonic development, organ formation, and fruit ripening. ARFs also play important roles in plant responses to both biotic and abiotic stresses [5,6]. Typically, ARF proteins consist of three functional domains: an N-terminal B3 DNA-binding domain (DBD), a middle region (MR), and a carboxyl-terminal domain (CTD). The B3 domain recognizes the auxin response element (AuxRE; TGTCTC) in the promoter regions of target genes, thereby facilitating DNA binding and regulating auxin-responsive gene expression. The MR determines the transcriptional regulatory activity of *ARFs*: glutamine-rich MRs recruit transcriptional coactivators to activate downstream gene expression, while proline-rich MRs impede the formation of the transcription initiation complex through repressor domains [7]. The CTD contains conserved motifs III and IV, which share structural similarity with those in Aux/IAA proteins [8]. ARF proteins can form complexes with Aux/IAAs via homo- or heterodimerization, thus modulating the expression of auxin-responsive genes [7].

The ARF gene family plays a crucial role in plant growth and development [4]. In Arabidopsis (*Arabidopsis thaliana* L.), *AtARF10* and *AtARF16* are highly expressed in the root cap and meristematic tissues, while *AtARF7* and *AtARF19* are involved in lateral root formation [9,10]. *AtARF5* and *AtARF8* coordinately regulate reproductive development, including stamen development, petal expansion, anther dehiscence, and pistil maturation [11,12]. In rice (*Oryza sativa* L.), *OsARF6* and *OsARF17* are involved in regulating flag leaf angle, *OsARF12* controls root cap and lateral root development, and *OsARF23* plays an essential role in the growth and development of seeds and vegetative organs [13,14]. In soybean (*Glycine max* (L.) Merr.), *GmARF8a* and *GmARF8b*, regulated by miR167, are critical for nodule formation and lateral root development [15]. Research in tomato (*Solanum lycopersicum* L.) also highlights the multifunctionality of *ARF* genes: *SlARF2*, *SlARF3*, *SlARF4*, *SlARF10* and *SlARF12* are involved in leaf morphogenesis, floral organ development, and fruit ripening [6,16,17].

Furthermore, importantly, the ARF gene family also plays a significant role in responses to abiotic stress. *AtARF10* and *AtARF16* are both targets of microRNA160. Auxin signaling can either activate or repress these genes, thereby regulating *ABI3* expression in the ABA signaling pathway to control seed dormancy and germination [18]. In tomato, salt stress results in the downregulation of most *SlARF* genes, while *SlARF1*, *SlARF4*, and *SlARF19* show significant upregulation [19]. In lettuce (*Lactuca sativa* L.), the expression of *LsARF* genes is closely associated with temperature changes, with *LsARF8a* involved in regulating bolting time [20]. Under drought and salt stress, *CqARF5*, *CqARF7*, *CqARF15*, and *CqARF24* in quinoa (*Chenopodium quinoa* Willd.) are highly expressed, and heterologous expression of *CqARF5* in Arabidopsis significantly enhances drought and salt tolerance in transgenic plants [21]. These studies collectively elucidate the diverse regulatory functions of *ARF* genes in plant growth, development, and stress response.

Sesame (*Sesamum indicum* L.) is a traditional oil crop in China, valued for its seeds rich in lipids, proteins, minerals, and antioxidants, which contribute to both nutritional and medicinal benefits [22]. As a dry-season crop, sesame is susceptible to abiotic stresses such as high temperature, drought, and soil salinization during germination. The ARF gene family, as key regulators in the auxin signaling pathway, has been extensively studied in more than a dozen plant species, including Arabidopsis, rice, soybean, tomato, maize (*Zea mays* L.), and sweet potato (*Ipomoea batatas* (L.) Lam.) [10,23,24,25,26]. However, its characterization in sesame remains largely unexplored. Although the seed germination stage is one of the most stress-sensitive phases in the plant life cycle, the expression patterns of *ARF* genes in sesame seedlings under high temperature, drought, and salt stress have not yet been reported. To address this knowledge gap, the present study provides the first comprehensive genome-wide identification of the *ARF* gene family in sesame. Using bioinformatics approaches, we systematically analyzed the physicochemical properties, conserved domains, gene structures, and cis-acting regulatory elements of *SiARF* genes, and we investigated the expression responses of *SiARF* genes to high temperature, drought, and salt stress during seed germination. These analyses fill a critical gap in the understanding of *ARF* genes in sesame and provide an important theoretical foundation for future studies aimed at elucidating the biological functions of *ARF* genes in sesame seed germination under abiotic stress conditions.

## 2. Results

### 2.1. Identification and Physicochemical Properties of the Sesame ARF Gene Family

Through homologous BLAST alignment and HMM domain searches using TBtools (TBtools-II, v2.390), 23 *ARF* genes were identified in the sesame genome, named *SiARF1* to *SiARF23* based on their linear chromosomal positions. The detailed characteristics of these ARF genes were presented in Table 1, including gene ID, protein length, molecular weight, pI, protein hydrophilicity, protein stability, and predicted subcellular localization. The results showed that *SiARF* proteins ranged from 516 to 1133 aa in length, with predicted molecular weights ranging from 56.57 to 126.07 kDa and theoretical pI values between 5.04 and 8.85. Subcellular localization and protein stability predictions indicated that all 23 SiARF proteins are predicted to localize to the nucleus and to be classified as unstable according to the instability index calculation. In addition, the hydrophilicity values of all proteins were less than 0, classifying them as hydrophilic proteins.

### 2.2. Chromosome Localization and Gene Duplication Analysis

**Figure 1:**
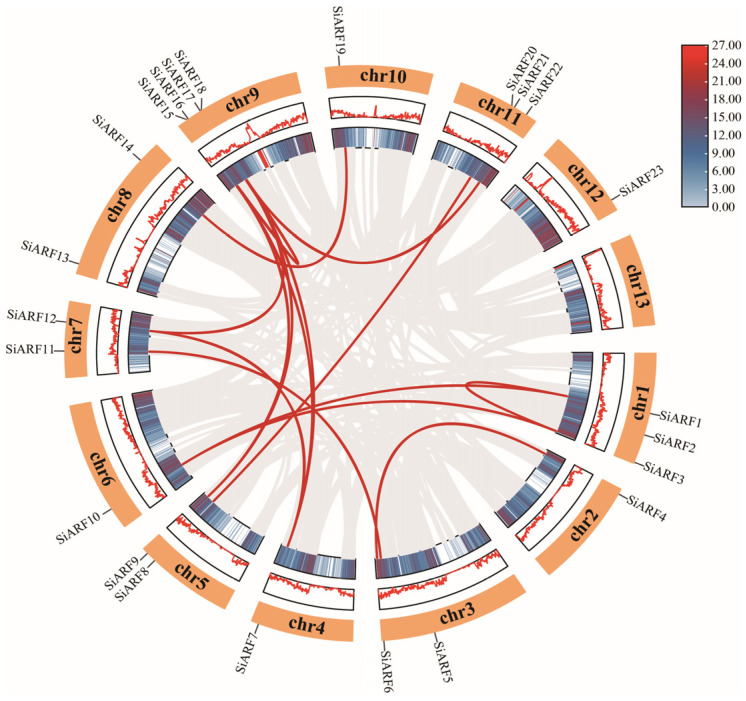
*SiARF* gene duplication on various chromosomes of sesame genome. Duplicated gene pairs are represented by red lines inside the circle. The orange-colored blocks in the figure represent chromosomes in the genome. The blue heat map and the broken red line map represent the gene density and expression levels of the *SiARF* gene, respectively.

The 23 *SiARF* genes were unevenly distributed across 12 sesame chromosomes (Chrs) (Figure S1). Specifically, Chr2, Chr4, Chr6, Chr10, and Chr12 each contained one *SiARF* gene, while Chr3, Chr5, Chr7, and Chr8 each contained two *SiARF* genes. Both Chr1 and Chr11 carried three *SiARF* genes, whereas Chr9 contained four. No *SiARF* genes were detected on Chr13 of the reference genome. Investigation of gene duplication events is crucial for understanding the evolution and expansion of the ARF gene family in sesame. The results revealed that 15 *SiARF* gene pairs were involved in segmental duplication, while no tandem duplication events were detected (Figure 1), suggesting that the *SiARF* gene family primarily expanded through segmental duplication. The gene-density track showed that *SiARF* genes were distributed in chromosomal regions with variable gene densities, suggesting that their locations were not limited to either gene-rich or gene-poor regions. In addition, the expression profile track revealed differences in transcript abundance among *SiARF* members, indicating that these genes may exhibit divergent expression patterns.

**Figure 2:**
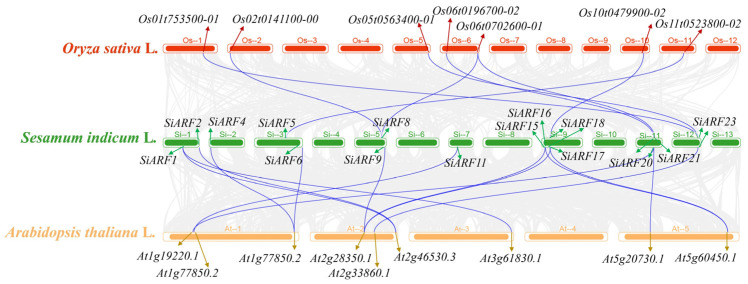
Comparative synteny analysis of *ARF* genes between sesame (green), and Arabidopsis (orange), rice (red). Blue lines represent syntenic *ARF* gene pairs between sesame and rice/Arabidopsis.

To further investigate the evolutionary relationships of the ARF gene family across different species, a whole-genome synteny analysis was performed among sesame, Arabidopsis, and rice. The results revealed 14 syntenic gene pairs between sesame and Arabidopsis, compared with only eight gene pairs between sesame and rice (Figure 2). This suggests a closer phylogenetic relationship and more extensive syntenic conservation between sesame and Arabidopsis than between sesame and rice, likely reflecting the evolutionary divergence between dicotyledonous and monocotyledonous species. Collinear gene pairs between sesame and Arabidopsis, and between sesame and rice, are listed in Table S5.

### 2.3. Phylogenetic Analysis

**Figure 3:**
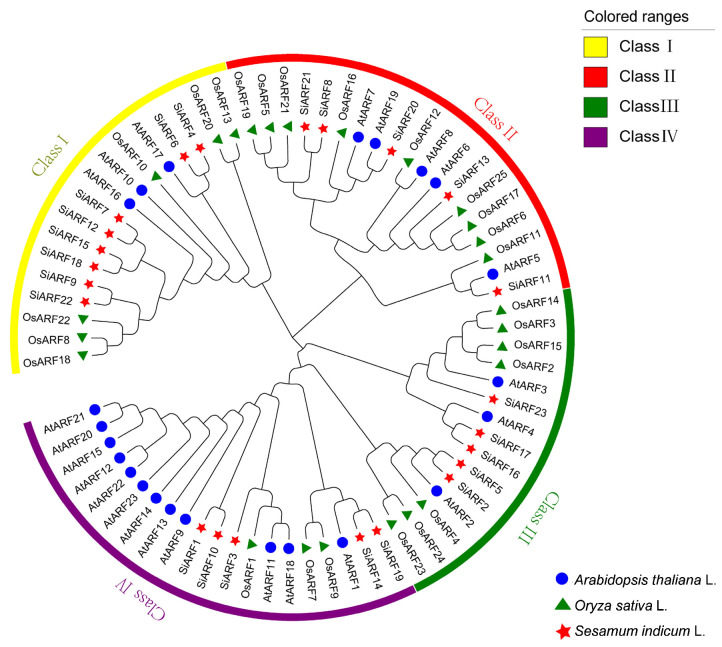
Phylogenetic tree of *ARFs* from sesame, rice and Arabidopsis. Note: The phylogenetic tree was constructed using MEGA-7.0 based on the maximum likelihood method; bootstrap repeats numbered 1000. Different subfamilies are highlighted in different colors. Blue solid circle, green solid triangle and red solid five-pointed star represent ARF protein from Arabidopsis, rice and sesame.

To investigate the phylogenetic relationships within the ARF gene family, a phylogenetic tree was constructed using MEGA 7.0 based on the ARF protein sequences from sesame, Arabidopsis, and rice. As shown in Figure 3, the ARF gene family was grouped into four distinct subfamilies (Class I–IV). Among these, Class I contained the largest number of *SiARF* members (eight members), followed by Class II (five members), Class III (three members), and Class IV (seven members) (Figure 3). The results suggest that the ARF gene family has undergone functional diversification during evolution, with each subfamily potentially carrying out distinct biological roles.

### 2.4. Gene Structure and Protein Conserved Motif Analysis

**Figure 4:**
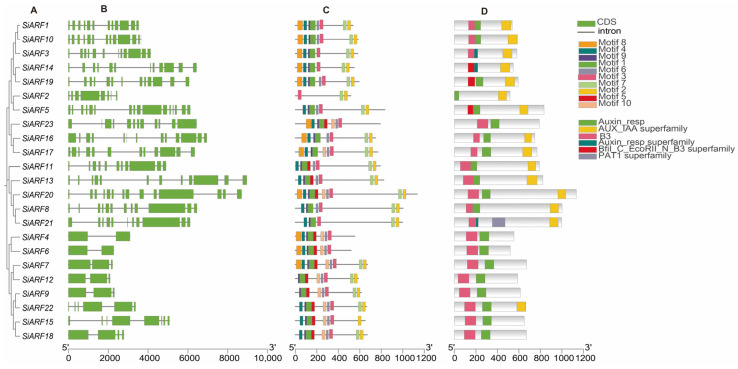
The conserved motifs, domains and gene structure of sesame *ARF* gene family. (**A**) Phylogenetic relationships. (**B**) Gene structure analysis. (**C**) Protein conservation motif analysis. (**D**) Protein conservation domain analysis.

All *SiARF* protein sequences contained the conserved auxin response domain (auxin_resp) and typical DBD (B3 domain) (Figure 4D). To further examine structural variations among *SiARF* proteins and to explore their functional, regulatory, and evolutionary features, gene structures were analyzed (Figure 4B). The results revealed considerable variation in sequence length and intron–exon organization among *SiARF* members. The *SiARF* genes contained between 2 and 16 coding sequences (CDSs) and between 1 and 15 introns, with no untranslated regions (UTRs) detected. Notably, the longest gene, *SiARF5*, contained 16 exons and 15 introns, while the shortest, *SiARF6*, contained only two exons and one intron.

Using the MEME online tool, 10 conserved motifs (Motif 1–10) were predicted in the *SiARF* protein sequences, with individual proteins containing between 3 and 10 motifs. Motifs within the same subfamily showed similar composition and positioning (Figure 4C). Motif 3 was present in all 23 *SiARF* proteins, suggesting it plays a fundamental functional role. Motifs 1, 4, 6, and 9 were also highly conserved among most *SiARF* members, indicating their evolutionary stability. Furthermore, members of the same subfamily, such as *SiARF1*, *SiARF10*, and *SiARF3*, shared similar motif patterns (Motifs 1/2/3/4/6/7/8/9), whereas members of different subfamilies, such as *SiARF4* and *SiARF16*, displayed significant motif divergence, implying potential functional specialization within the *SiARF* family.

### 2.5. Analysis of Cis-Acting Regulatory Elements of SiARFs

Cis-acting regulatory elements serve as crucial molecular switches for transcriptional regulation, governing dynamic networks of gene expression involved in various biological processes. To explore the regulatory expression patterns and potential functional roles of *SiARF* genes, the 2000 bp sequences upstream of their translation start codons were extracted and analyzed using the PlantCARE online tool. A total of 44 types of response elements were identified, related to light, phytohormones, abiotic stress, and plant growth and development (Table S4).

**Figure 5:**
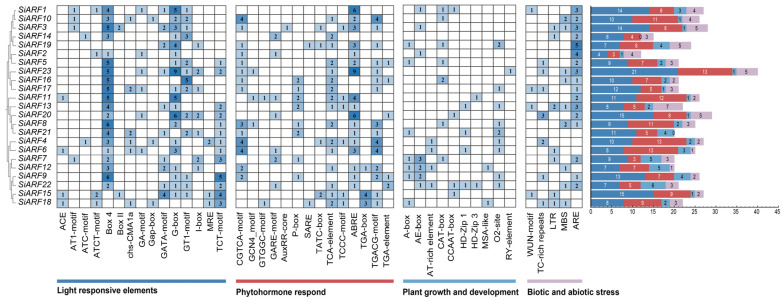
Predicted cis-elements in the promoter regions of the *SiARF* genes. The bar graph on the right displays the number of different types of cis-acting elements within the 2 kb upstream region of each *SiARF* gene.

All *SiARF* genes contained light-responsive elements, which were the most abundant, suggesting that the gene expression of *SiARFs* may be strongly influenced by light (Figure 5). Hormone-related elements included those responsive to auxin, GA, ABA, and methyl jasmonate (MeJA). Specifically, 23 genes contained ABA-responsive elements, 16 genes harbored MeJA-responsive elements, 12 genes possessed GA-responsive elements, and 7 genes carried auxin-responsive elements.

Most *SiARF* genes contained ABRE (ABA-responsive element), TCA-element, and TGACG-motif. Notably, nine ABRE elements were identified in the promoter of *SiARF23*, while *SiARF1*, *SiARF11*, and *SiARF20* contained six, four, and six ABREs, respectively. Since ABA is closely associated with drought resistance, these genes (*SiARF1*, *SiARF11*, *SiARF20*, and *SiARF23*) may play important roles in plant drought tolerance.

Among abiotic stress-related elements, anaerobic induction elements were the most widely distributed, with 82.61% of *SiARF* genes containing at least one such element. In addition, wound-responsive elements, defense and stress-responsive elements, low temperature-responsive elements, and drought-inducible elements were detected in the promoters of *SiARF1*, *SiARF4*, *SiARF12*, and *SiARF14*, respectively. The MYB-binding site (MBS), which is strongly linked to drought responsiveness, was found in the promoters of *SiARF8*, *SiARF10*, and *SiARF22*, each containing one or two MBS elements, suggesting their involvement in drought resistance.

Furthermore, some tissue-specific regulatory elements were identified, including a seed-specific regulatory element exclusively present in *SiARF23*. The diverse array of cis-acting elements found in the promoter regions of *SiARF* genes indicates that the ARF gene family in sesame is likely regulated by multiple phytohormones and involved in various abiotic stress response pathways.

### 2.6. Tissue-Specific Expression Profiling of SiARFs

**Figure 6:**
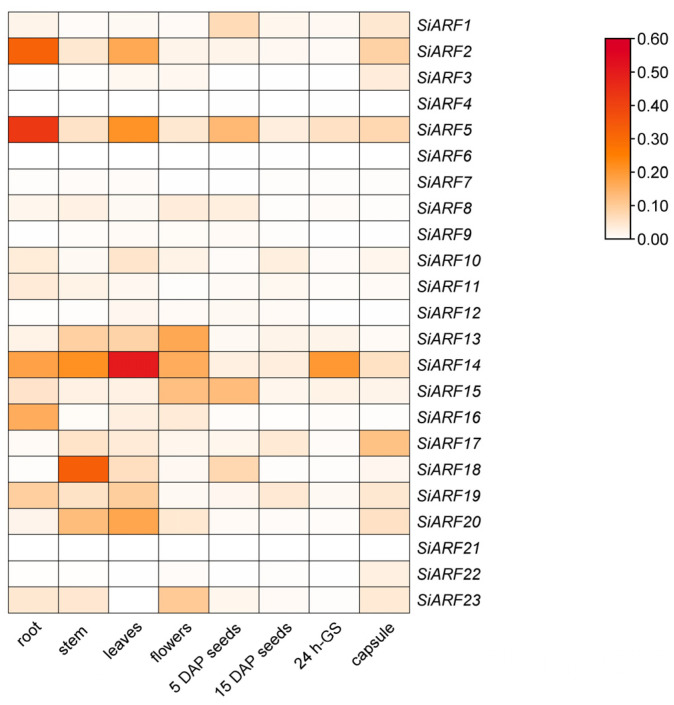
Heatmap of relative expression levels of the *SiARF* genes in different tissues. The relative expression data was obtained from qRT–PCR calculated by using *SiUBQ6* as the internal reference gene. DAP: days after pollination, GS: germinated seeds.

Tissue-specific expression of *SiARF* genes is associated with their specialized functions in particular organs. To investigate the expression patterns of *SiARF* genes across various tissues and organs in sesame, transcript levels were analyzed using qRT-PCR under normal growth conditions. Eight different tissues were examined: root, stem, leaf, flower, capsule, 5- days after pollination (DAP) seeds, 15-DAP seeds, and 24 h germinated seeds (24h-GS) (Figure 6, Table S2).

Notably, *SiARF14* exhibited high expression in roots, stems, leaves, and 24h-GS, with the highest transcript abundance observed in germinated seeds, suggesting a potential role in seed development. *SiARF5* was highly expressed in roots, stems, and 24h-GS, with its highest expression in roots, indicating its functional importance in root development. *SiARF2* showed elevated expression in roots and leaves, while *SiARF18* was predominantly expressed in stems, implying its involvement in stem development. *SiARF16* was primarily expressed in roots; *SiARF17* in stems and leaves; *SiARF18* in capsules; and *SiARF19* in roots, stems, and leaves; *SiARF23* in flowers.

In contrast, ten *SiARF* genes (*SiARF1*, *SiARF3*, *SiARF4*, *SiARF6*, *SiARF7*, *SiARF9*, *SiARF11*, *SiARF12*, *SiARF21* and *SiARF22*) exhibited relatively low expression levels across all eight tissues. The divergent expression profiles of the *SiARF* gene family members across sesame tissues provide valuable insights for further elucidation of their functional roles in sesame growth and development.

### 2.7. Expression Analysis of SiARFs Under Abiotic Stress

**Figure 7:**
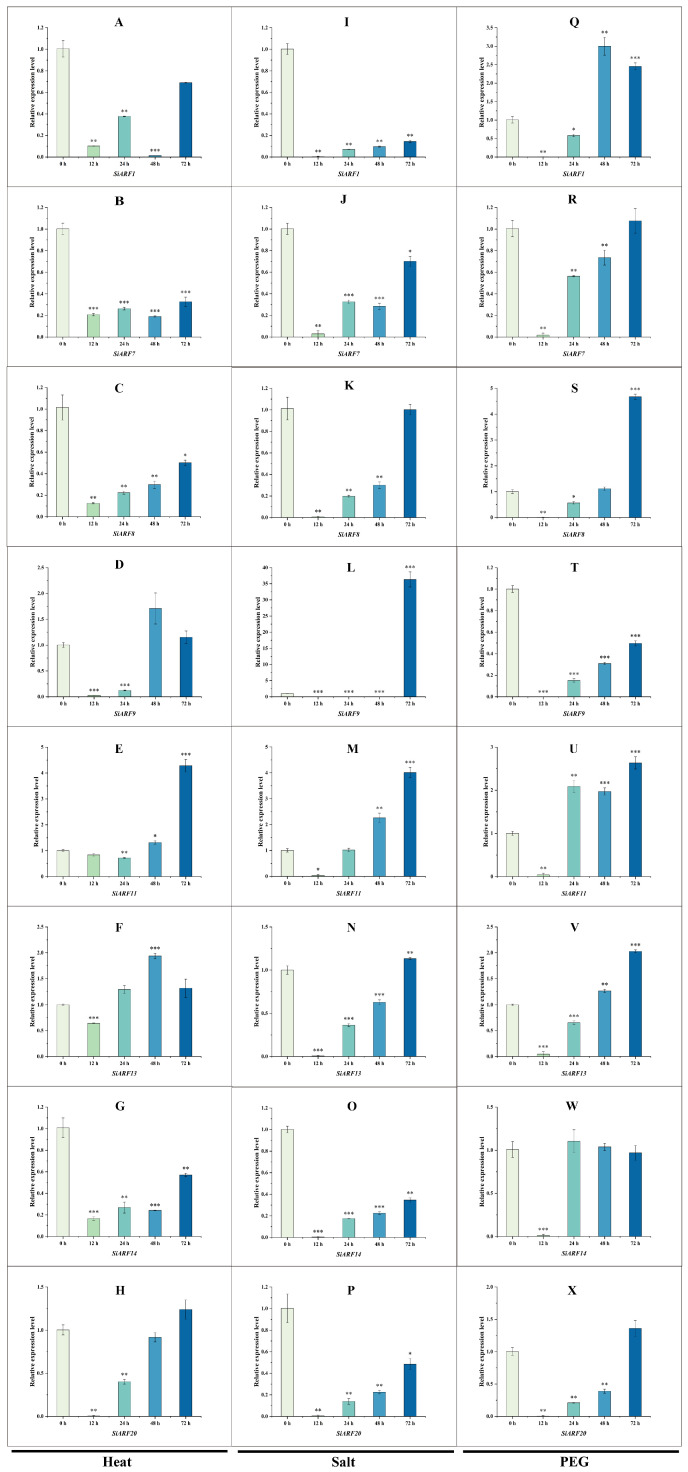
The relative expression levels of 8 selected *SiARFs* under abiotic stress as revealed by qRT–PCR. (**A**–**H**) The relative expression levels of selected genes under heat treatment; (**I**–**P**) the relative expression levels of selected genes under salt treatment; (**Q**–**X**) the relative expression levels of selected genes under drought treatment. Statistical significance was determined using *t*-tests (*** *p* < 0.001, ** *p* < 0.01, * *p* < 0.05, *n* = 3).

To preliminarily assess the expression patterns of *SiARF* genes under abiotic stress conditions, eight genes were selected for qRT-PCR analysis based on phylogenetic classification and cis-acting element prediction (Figure S2, Table S3). Under high-temperature stress (Figure 7A–H), *SiARF9* showed upregulated expression at 48 and 72 h, while *SiARF13* was upregulated at 24, 48, and 72 h. *SiARF20* exhibited increased expression at 72 h, and *SiARF11* reached peak expression (4.3-fold increase) at 72 h. The remaining genes were downregulated. Under salt stress (Figure 7I–P), *SiARF11* was significantly upregulated at 24 and 48 h (2.3-fold peak) and continued to increase at 72 h (4-fold peak). *SiARF13* was also upregulated at 72 h. Notably, *SiARF9* showed a dramatic 36-fold increase in transcript level relative to the control at 72 h, indicating a possible key regulatory role in the salt stress response. The remaining genes were downregulated under salt treatment. During drought stress (Figure 7Q–X), four genes, *SiARF1*, *SiARF8*, *SiARF11*, and *SiARF13*, exhibited consistent upregulation at multiple time points. *SiARF1* reached peak expression at 48 h (3.0-fold) and remained elevated at 72 h (2.4-fold). *SiARF8* displayed a maximum 4.6-fold increase at 72 h. *SiARF11* showed elevated levels at 24 h (2.08-fold), 48 h (1.97-fold), and 72 h (2.63-fold). Additionally, *SiARF7*, *SiARF14*, and *SiARF20* were upregulated at 72, 24, and 72 h, respectively.

In summary, several *SiARF* genes exhibited similar expression trends under different abiotic stress conditions. *SiARF9*, *SiARF11*, and *SiARF13* were upregulated at 72 h under both high-temperature and salt stress, while *SiARF1*, *SiARF7*, *SiARF8*, and *SiARF14* were generally downregulated. These findings suggest that specific *SiARF* genes may play critical roles in the response to multiple abiotic stresses in sesame.

## 3. Discussion

### 3.1. Characterization of the Sesame ARF Gene Family

In recent years, the ARF family has been identified in numerous plant species, including Arabidopsis, rice, and tomato. This study presents the first genome-wide identification and characterization of the *ARF* gene family in sesame. A total of 23 *ARF* members were identified, a number comparable to those reported in Arabidopsis (23), rice (25), and tomato (22) [6,23,27]. Previous studies have shown that ARF proteins generally contain conserved functional domains, including an N-terminal DNA-binding domain, a middle region, and a C-terminal PB1/interaction domain, which are essential for regulating auxin-mediated transcriptional responses [7,8]. Therefore, the comparable size of the *ARF* gene family in sesame and other plants suggests that this family has been relatively conserved during plant evolution to maintain fundamental auxin signaling functions. The 23 SiARF proteins range in length from 516 to 1133 aa, with molecular weights between 56.57 and 126.07 kDa and pI ranging from 5.04 to 8.86. The instability indices of all proteins exceeded the standard threshold of 40 (ranging from 42.92 to 69.81), indicating that they are predicted to be unstable. The grand average of hydropathicity (GRAVY) values ranged from −0.295 to −0.701, all negative, suggesting that the *SiARF* proteins are hydrophilic. These results indicate that SiARF proteins possess strong hydrophilicity. As transcription factors, ARF proteins typically function in the nucleus. Subcellular localization prediction analysis confirmed that all 23 SiARF proteins are predicted to localize in the nucleus (Table 1), consistent with findings reported in flax (*Linum usitatissimum* L.) [28], tomato [6], and maize [24].

The 23 *SiARF* genes were found to be unevenly distributed across the 12 chromosomes of sesame. Gene duplication is a key driver of genome evolution and functional diversification. Most gene families expand through tandem and/or segmental duplication, with the latter being a major mechanism of expansion [29]. In this study, 15 segmentally duplicated *SiARF* gene pairs were identified, and no tandem duplications were detected, consistent with findings in rice and Arabidopsis [23,27]. Collinearity analysis provides insight into genome structure and phylogenetic relationships, which reflect conserved gene order and chromosomal positioning within and across genomes. In this study, a total of 22 collinear gene pairs were identified between sesame and Arabidopsis/rice, involving 15 *SiARF* genes (65.22% of the 23 ARF family members), demonstrating the evolutionary conservation of *ARF* genes in sesame. Domain analysis revealed that all 23 *SiARF* proteins possess the conserved Auxin_resp domain, consistent with domain structures reported in wheat (*Triticum aestivum* L.) [30], tomato [6], soybean [26], and other species. Motif analysis showed that Motif 3 is highly conserved across the *SiARF* gene family. Members within the same subfamily, such as *SiARF1*, *SiARF10*, and *SiARF3*, shared similar motif patterns (Motifs 8/4/9/1/6/3/7/2), while members of different subfamilies, such as *SiARF4* and *SiARF16*, exhibited distinct motif compositions, indicating possible functional divergence. Gene structure analysis revealed that *SiARF* genes contain between one and 15 CDSs, with no UTRs detected. This structural simplicity is consistent with previous observations in papaya (*Carica papaya* L.) [31], apple (*Malus domestica* Borkh.) [32], maize [24], and wheat [33], suggesting that the *ARF* gene family generally exhibits a straightforward gene structure.

Furthermore, further classification of cis-acting regulatory elements revealed that the promoters of *SiARF* genes contain multiple regulatory elements associated with light responsiveness, phytohormone signal transduction, plant growth and development, and biotic and abiotic stress responses. The relatively abundant distribution of light- and hormone-responsive elements suggests that *SiARF* genes may be co-regulated by environmental cues and multiple hormonal pathways. Meanwhile, the presence of development-related and stress-responsive elements indicates that different *SiARF* members may participate in distinct developmental processes and stress adaptation. Differences in the composition of cis-acting elements among promoters may provide a potential regulatory basis for the functional diversification of *SiARF* genes [34,35].

### 3.2. Phylogenetic Characterization and Function of the ARF Gene Family

A phylogenetic tree was constructed to analyze the evolutionary relationships of ARF gene family members between sesame and Arabidopsis, and between sesame and rice (Figure 3). The *ARF* gene family is classified into four subfamilies, and genes within closer phylogenetic clades are more likely to share similar functions. Class I includes eight *SiARF* genes (*SiARF4*, *SiARF6*, *SiARF7*, *SiARF9*, *SiARF12*, *SiARF15*, *SiARF18*, and *SiARF22*) and three Arabidopsis genes (*AtARF10*, *AtARF16*, and *AtARF17*). Previous studies in Arabidopsis have shown that *AtARF10* and *AtARF16* are target genes of microRNA160. Auxin signaling modulates the activation or repression of these genes, thereby regulating the expression of *ABI3* in the ABA signaling pathway, which controls seed dormancy and germination [18]. Cis-acting element analysis revealed that *SiARF7*, *SiARF9*, *SiARF18*, and *SiARF22* contain elements responsive to auxin, GA, and ABA, such as ARE, GARE-motif, TATC-box, and ABRE. Phylogenetic analysis shows that *SiARF7*, *SiARF9*, *SiARF18*, and *SiARF22* are closely related to *AtARF10* and *AtARF16*, suggesting that these sesame *ARF* genes may play key regulatory roles in response to abiotic stress during seed germination.

Class II comprises five *SiARF* genes and five *AtARF* genes (*AtARF5*, *AtARF6*, *AtARF7*, *AtARF8*, and *AtARF19*. *AtARF5*, *AtARF6*, *AtARF7*, and *AtARF8* exhibit distinct expression patterns in different tissues. *IAA12* and *AtARF5* interact to regulate root meristem initiation during early embryo germination [35,36]. *AtARF6* and *AtARF8*, targets of miR167, are essential for embryonic development [37,38]. Furthermore, *AtARF6* interacts with transcription factors *BZR1* and *PIF4* to coordinate responses to hormonal signals (GA, BR, IAA) and environmental cues (light, temperature), promoting hypocotyl elongation during seed germination and seedling establishment. *BZR1* and *PIF4* also interact specifically with *AtARF8* to regulate hypocotyl or shoot organ development [39,40]. Class III includes three sesame genes that are closely related to *AtARF3* and *AtARF4*. Clade IV contains seven sesame genes that are more closely related to 13 Arabidopsis genes, including *AtARF1*, *AtARF2*, and *AtARF11*. *AtARF2* acts as an auxin repressor and participates in the ABA pathway to regulate seed germination [36]. Cis-acting element analysis showed that, except for *SiARF14*, the other six genes in Clade IV contain one to five ABREs, suggesting that these *SiARF* genes may function as auxin repressors in the ABA pathway to regulate seed germination.

### 3.3. Expression Analysis of the Sesame ARF Gene Family

The *auxin* gene family plays key roles in plant growth and development. In this study, we examined the expression profiles of *ARF* gene family members in sesame across different tissues using qRT-PCR. Among all members, *SiARF14* exhibited the highest expression levels in three tissues (stem, leaf, and 24h-GS), suggesting its potential positive regulatory role in sesame growth. *SiARF2* showed relatively high expression in roots, leaves, and capsules. *SiARF5* was highly expressed in roots, leaves, and 5-DAP seeds. *SiARF17* was highly expressed in stems and leaves. *SiARF19* was highly expressed in roots, stems and leaves. *SiARF23* was highly expressed in flowers. *SiARF18* exhibited elevated expression in stems. *SiARF13* was highly expressed in flowers but showed extremely low expression in capsules. The remaining *SiARF* genes exhibited low expression levels in all tissues tested. The spatiotemporal variation in expression patterns among *SiARFs* suggests that different members of the sesame *ARF* gene family may have distinct biological functions. Furthermore, most *SiARF* genes were found to contain cis-acting elements associated with stress, hormone signaling, growth and development, and light response, such as AUXRE, ABRE, GARE-motif, TATA-box, and ACE. The discovery of these regulatory elements indicates that *ARF* genes may participate in responses to abiotic stress through multiple cis-regulatory pathways.

Auxin is involved in nearly all developmental stages of plants, from seed germination to senescence. Seed germination is critical in determining biomass and yield in later growth stages. With the increasing frequency of extreme climatic events, plants are increasingly exposed to adverse conditions such as drought, high salinity, and elevated temperatures [6,21,41]. Previous studies have shown that *ARF* genes function as transcriptional regulators in auxin signal transduction and stress response [42]. In rice, *OsARF17* regulates seed development through the AET1-mediated pathway and contributes to high-temperature tolerance [43]. In sugar beet (*Beta vulgaris* L.), drought stress significantly induces *EntrezID-104908672* and *EntrezID-104903260*, particularly during the transition from testa-rupture to radicle protrusion, thereby improving drought tolerance during germination [44]. In alfalfa (*Medicago sativa* L.), *MsARF039* and *MsARF5* positively regulate heat and cold stress responses [45]. In maize, *ZmARF5* and *ZmARF22* are significantly upregulated under salt stress [24]. In tomato, *SlARF1*, *SlARF4*, and *SlARF6B* show marked upregulation under drought conditions [19]. In this study, eight sesame *ARF* genes were selected to analyze their expression profiles under high-temperature, salt, and drought stresses. The results revealed that *SiARF11* and *SiARF13* were significantly upregulated under all three stress conditions. *SiARF11* was gradually upregulated with increasing stress duration under heat, drought, and salt stress conditions, indicating that it may represent a key broad-spectrum stress-responsive ARF gene. Previous studies have shown that *OsARF11* in rice and multiple ARF members in tomato and potato are involved in responses to salt, drought, and other abiotic stresses [19,46,47]. Accordingly, *SiARF11* may participate in coordinating stress adaptation and growth regulation in sesame through the ARF-mediated auxin signaling pathway.

Notably, *SiARF8* exhibited a 4.6-fold increase (*p* < 0.001) under drought stress at 72 h, suggesting a critical role in drought tolerance. Remarkably, *SiARF9* showed a dramatic 36-fold upregulation (*p* < 0.001) after 72 h of salt stress. This pronounced upregulation suggests that *SiARF9* may be a candidate ARF gene involved in salt stress response during the late stage of sesame seed germination. Similar salt-responsive *ARF* genes have been reported in other species, including tomato, rice, and sweetpotato, indicating that ARF-mediated auxin signaling may participate in plant adaptation to salt stress [19,46,48]. Salt stress typically induces ABA accumulation and affects auxin distribution and signaling. Accordingly, we hypothesize that the strong upregulation of *SiARF9* may be involved in signal crosstalk between auxin-dependent transcriptional regulation and ABA-mediated stress adaptation [49,50]. In this process, *SiARF9* may influence the expression of downstream genes involved in growth regulation, osmotic balance, reactive oxygen species (ROS) scavenging, and stress-related hormone signaling, thereby contributing to the restoration of cellular homeostasis and potentially enhancing salt stress tolerance.

Further analysis showed that most of the selected *SiARF* genes displayed biphasic expression patterns under high-temperature, drought, and salt stress: an initial downregulation in the early phase (0–24 h), followed by progressive upregulation from 24 to 72 h (Figure 7). These time-dependent expression patterns are closely associated with the stress adaptation process during seed germination. The widespread downregulation of *SiARFs* during the early stress phase may reflect a transient suppression of auxin signaling, potentially mediated by rapid activation of abscisic acid (ABA) signaling, with the biological significance of inhibiting germination and conserving energy under unfavorable conditions [51,52]. In contrast, the gradual upregulation of *SiARFs* during the late stress phase may indicate a reactivation of auxin signaling, contributing to the establishment of cellular homeostasis and the enhancement of stress adaptation [52,53].

Notably, the promoter regions of multiple stress-responsive *SiARF* genes (including *SiARF8*, *SiARF9*, *SiARF11*, and *SiARF13*) contain stress-related cis-elements such as ABRE, MBS, and ARE. The presence of these cis-elements provides a molecular basis for the direct regulation of *SiARF* expression by ABA signaling [49,50,51]. Accordingly, we hypothesize that under drought or salt stress, elevated endogenous ABA levels activate ABF/AREB-type bZIP transcription factors, which subsequently bind to ABREs in *SiARF* promoters to dynamically modulate their transcriptional activity [54,55]. This regulatory mechanism may serve as a molecular bridge between ARF-mediated auxin signaling and ABA-dependent stress signaling, with the two pathways acting antagonistically or synergistically to coordinate stress adaptive responses during seed germination [52,56]. These findings provide a theoretical basis for exploring the application of *SiARF* genes in the breeding of stress-tolerant crop varieties. Based on these findings, *SiARF11* and *SiARF9* were preliminarily identified as candidate genes associated with stress tolerance in this study, and their specific roles in stress adaptation warrant further investigation through functional validation experiments.

## 4. Materials and Methods

### 4.1. Identification of the ARF Gene Family in Sesame

The genomic sequence and annotation information for sesame were obtained from https://doi.org/10.6084/m9.figshare.21151948. The protein sequences of Arabidopsis ARFs were downloaded from the Ensembl Plants database (https://www.ensembl.org, accessed on 16 January 2025). Hidden Markov Model (HMM) profile files (PF02362 and PF06507) were retrieved from the Pfam database (http://pfam.xfam.org/, accessed on 16 January 2025). The *SiARF* family proteins in the sesame genome were identified using BLASTp and HMMER 3.0, with an E-value threshold of 1e^−5^. The presence of conserved domains characteristic of the ARF protein family was confirmed using InterPro (https://www.ebi.ac.uk/interpro/, accessed on 16 January 2025) and NCBI-CDD (https://www.ncbi.nlm.nih.gov/Structure/bwrpsb/bwrpsb.cgi, accessed on 17 January 2025). Proteins lacking typical ARF domains were excluded.

The molecular weight, isoelectric point (pI), amino acid (aa) composition, hydrophobicity, and instability index of the sesame ARF proteins were predicted using the ExPASy online platform (https://www.expasy.org, accessed on 20 January 2025). Subcellular localization predictions for sesame ARF proteins were performed using WoLF PSORT (https://wolfpsort.hgc.jp/, accessed on 10 February 2025). Additionally, the chromosomal localization of sesame ARF genes was analyzed using the “Genes Location Visualize (Advanced)” plugin in TBtools.

### 4.2. Phylogenetic Analysis, Conserved Motifs, Gene Structure, and Synteny Analysis

Multiple sequence alignments of ARF proteins from sesame, Arabidopsis, and rice were performed using Clustal W in MEGA 7.0. A phylogenetic tree was constructed using the maximum likelihood method, and the resulting tree was refined and visualized using the online tool iTOL (https://itol.embl.de/, accessed on 17 February 2025). Conserved motifs within sesame ARF proteins were identified using the MEME online tool (https://meme-suite.org/meme/tools/meme, accessed on 17 February 2025), with the number of motifs set to 10 and all other parameters set to default values. Conserved protein domains of *SiARF* members were further confirmed through the NCBI-CDD online database. Intron and exon structures of the sesame ARF genes were extracted using TBtools, and gene structure information was visualized using the “Genes Location Visualize (Advanced)” function in TBtools. In addition, synteny analysis among *SiARF*, *AtARF*, and *OsARF* genes was performed using McScanX within TBtools, and both intra- and inter-species synteny maps were generated.

### 4.3. Analysis of Cis-Acting Elements

TBtools software (TBtools-II, v2.390) was used to extract the 2000 bp upstream sequences from the start codon of the *SiARF* genes. These sequences were submitted to the PlantCARE online tool (https://bioinformatics.psb.ugent.be/webtools/plantcare/html/, accessed on 20 March 2025) to identify cis-acting elements in the promoter regions. The types, distribution, and predicted functions of the cis-acting elements were statistically analyzed and categorized to elucidate their potential regulatory roles.

### 4.4. Plant Material Growth and Stress Treatment

The plant material used in this study was the sesame cultivar ‘Zhongzhi 13’. Plump seeds were selected and sown in a field experimental plot with a row spacing of 40 cm and plant spacing of 15 cm. Samples were collected at 5, 15, and 25 days after pollination (DAP), as well as from flower buds and 25-day-old capsule tissues. Root, stem, and leaf tissues were harvested from 2-week-old seedlings grown in 1/2 Hoagland nutrient solution under controlled growth chamber conditions at 28 °C, with a photoperiod of 16 h light and 8 h dark.

To investigate the response mechanisms of *SiARF* genes during seed germination under abiotic stress, three treatments were applied: drought, salinity, and high temperature. For the high-temperature treatment, sesame seeds were sown in germination boxes and placed in an incubator set at 44 °C. For drought and salt treatments, seeds were sown in germination boxes containing either 150 mmol/L NaCl (salt stress) or 15% PEG 6000 (drought stress). Germinated seed samples were collected at 0, 12, 24, 48, and 72 h post-treatment [57,58,59]. All samples were immediately flash-frozen in liquid nitrogen and stored at −80 °C for subsequent analyses. Samples of each treatment group included three biological replicates to ensure reproducibility.

### 4.5. Total RNA Extraction and Quantitative Polymerase Chain Reaction Analysis

Total RNA was extracted from all samples using the FastPure Universal Plant Total RNA Isolation Kit (RC411, Vazyme, Nanjing, China). First-strand cDNA synthesis was performed using the HiScript III All-in-One RT SuperMix Perfect for qPCR (R333, Vazyme, Nanjing, China) for quantitative reverse transcription polymerase chain reaction (qRT-PCR). qPCR was conducted using LightCycler 96 (Roche Molecular Systems, Pleasanton, CA, USA) and ChamQ Universal SYBR qPCR Master Mix (Q711, Vazyme, Nanjing, China). The qRT-PCR program was set as follows: pre-denaturation at 95 °C for 30 s (1 cycle); 40 cycles of 95 °C for 10 s and 60 °C for 30 s; followed by a melting curve step at 65 °C for 15 s. For sesame tissue samples, three biological replicates were analyzed. For abiotic stress-treated samples (heat, salt and drought stress), based on phylogenetic classification and cis-acting element prediction, eight genes selected from 23 *ARF* genes were subjected to three biological replicates, each with three technical replicates, and the arithmetic mean of the three technical replicates was used for statistical analysis. Student’s *t*-test was performed using Origin 2021 software (OriginLab Corporation, Northampton, MA, USA) to compare the treatment and control groups, with the significance level set at *p* < 0.05, and graphs were generated using the same software. The *SiUBQ6* gene was used as the internal reference [60]. Relative gene expression levels under abiotic stress were calculated using the 2^−ΔΔCT^ method, and expression levels in different tissues were determined using the 2^−ΔCT^ method [61]. Primers were designed using Primer Premier 6 and synthesized by Sangon Biotech (Shanghai, China) Co., Ltd. All primer sequences are listed in Table S1.

## 5. Conclusions

In summary, this study identified 23 *ARF* genes in sesame and classified them into four subfamilies. Genes within the same subfamily typically share similar gene structures and conserved functional domains. The characterization of the *SiARF* gene family included analyses of physicochemical properties, phylogenetic relationships, conserved motifs, gene structure, cis-acting elements, chromosomal distribution, subcellular localization prediction, and tissue-specific expression. Additionally, we examined the expression profiles of the selected *ARF* genes under high-temperature and salt- and drought-stress conditions and identified *SiARF11* and *SiARF9* as stress-responsive candidates. *SiARF11* was significantly induced under all three stress conditions, while *SiARF9* exhibited strong upregulation specifically under salt stress. This study lays a foundation for elucidating the functional roles and molecular mechanisms of *auxin* genes in plant development and abiotic stress responses, and contributes to the future development of stress-resilient sesame cultivars.

## Figures and Tables

**Table 1:** Basic information on Auxin family members in sesame.

Name	Gene ID	Gene Localization	Protein Length (aa)	MW(KD)	PI	GRAVY ^1^	II ^2^	PSL ^3^
*SiARF1*	*Sesame00594.t1*	Chr1:12,749,606:12,753,130	537	60.19	5.26	−0.602	58.87	Nucleus.
*SiARF2*	*Sesame01173.t1*	Chr1:17,410,846:17,413,290	516	58.03	6.14	−0.701	63.35	Nucleus.
*SiARF3*	*Sesame01945.t1*	Chr1:23,494,267:23,498,389	580	64.49	5.47	−0.513	50.95	Nucleus.
*SiARF4*	*Sesame02155.t1*	Chr2:1,127,569:1,130,658	553	60.71	6.25	−0.370	54.74	Nucleus.
*SiARF5*	*Sesame04963.t1*	Chr3:20,518,642:20,524,756	833	92.53	6.19	−0.530	59.36	Nucleus.
*SiARF6*	*Sesame06024.t1*	Chr3:31,059,341:31,061,614	518	56.57	8.42	−0.397	42.92	Nucleus.
*SiARF7*	*Sesame07469.t1*	Chr4:19,662,842:19,665,055	671	74.06	6.2	−0.387	43.90	Nucleus.
*SiARF8*	*Sesame08426.t1*	Chr5:17,606,168:17,612,616	1001	110.33	5.77	−0.585	63.79	Nucleus.
*SiARF9*	*Sesame08662.t1*	Chr5:19,698,545:19,700,851	612	67.45	6.34	−0.416	47.58	Nucleus.
*SiARF10*	*Sesame09303.t1*	Chr6:4,615,256:4,618,897	585	65.32	5.75	−0.648	61.57	Nucleus.
*SiARF11*	*Sesame11450.t1*	Chr7:5,602,499:5,607,697	790	87.50	5.04	−0.459	49.10	Nucleus.
*SiARF12*	*Sesame12012.t1*	Chr7:11,700,706:11,702,796	587	64.74	6.5	−0.381	50.58	Nucleus.
*SiARF13*	*Sesame12807.t1*	Chr8:2,060,688:2,069,641	822	91.46	5.75	−0.521	59.22	Nucleus.
*SiARF14*	*Sesame14350.t1*	Chr8:27,392,260:27,398,691	547	60.91	5.15	−0.568	67.87	Nucleus.
*SiARF15*	*Sesame15337.t1*	Chr9:2,216,355:2,221,422	649	70.50	8.86	−0.374	44.46	Nucleus.
*SiARF16*	*Sesame15363.t1*	Chr9:2,439,032:2,445,976	747	82.57	5.43	−0.463	51.61	Nucleus.
*SiARF17*	*Sesame15745.t1*	Chr9:5,413,016:5,419,352	768	85.35	5.9	−0.490	53.83	Nucleus.
*SiARF18*	*Sesame15766.t1*	Chr9:5,621,079:5,623,859	668	73.44	7.93	−0.295	46.26	Nucleus.
*SiARF19*	*Sesame18139.t1*	Chr10:2,916,794:2,922,852	592	66.41	5.12	−0.583	69.81	Nucleus.
*SiARF20*	*Sesame19944.t1*	Chr11:11,520,322:11,529,016	1133	126.07	6.23	−0.546	62.79	Nucleus.
*SiARF21*	*Sesame20005.t1*	Chr11:12,087,992:12,094,099	997	110.28	5.41	−0.588	63.58	Nucleus.
*SiARF22*	*Sesame20329.t1*	Chr11:14,980,971:14,984,337	663	72.39	6.62	−0.310	51.78	Nucleus.
*SiARF23*	*Sesame22207.t1*	Chr12:16,759,035:16,765,466	789	86.77	7.87	−0.384	52.15	Nucleus.

^1^ GRAVY: Grand Average of Hydropathicity; ^2^ II: Instability index; ^3^ PSL: Predicted Subcellular localization.

## Data Availability

All data are contained within the article or Supplementary Materials.

## Supplementary Materials

The following supporting information can be downloaded at: https://www.mdpi.com/article/10.3390/ijms27125470/s1.

## Abbreviations

The following abbreviations are used in this manuscript:

ARFs	Auxin response factors
ABA	Abscisic acid
GA	Gibberellins auxin
BR	Brassinosteroids
Aux/IAAs	AUX/indole-3-acetic acid
BDB	B3DNA-binding domain
MR	Middle region
CTD	Carboxyl-terminal domain
DAP	Days after pollination
GS	Germinated seeds
GRAVY	Grand Average of Hydropathicity
II	Instability index
PSL	Predicted subcellular localization
qRT-PCR	Quantitative reverse transcription polymerase chain reaction
