# Supplementary figures and images for: Genome-Wide Identification and Expression Profiling of the ARF Gene Family During Seed Germination in Sesame (Sesamum indicum L.) Under Abiotic Stresses
Source: Int J Mol Sci. 2026 Jun 17;27(12):5470. doi: 10.3390/ijms27125470 (PMC13300585; doi:10.3390/ijms27125470)

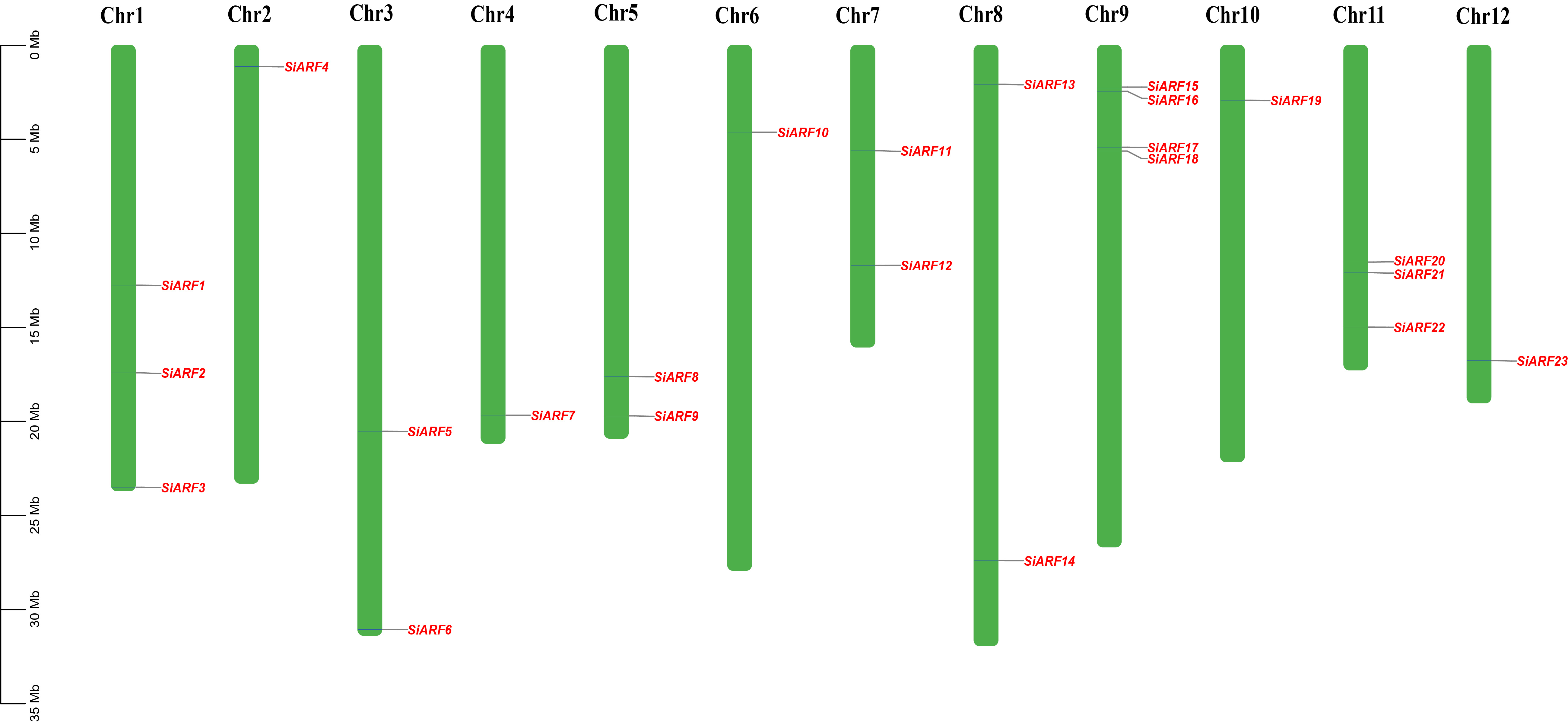

Supplement: Supplementary file 1 [file ijms-27-05470-s001.zip › Figure S1. Distribution of SiARF genes within the sesame chromosome (Chr). .jpg]

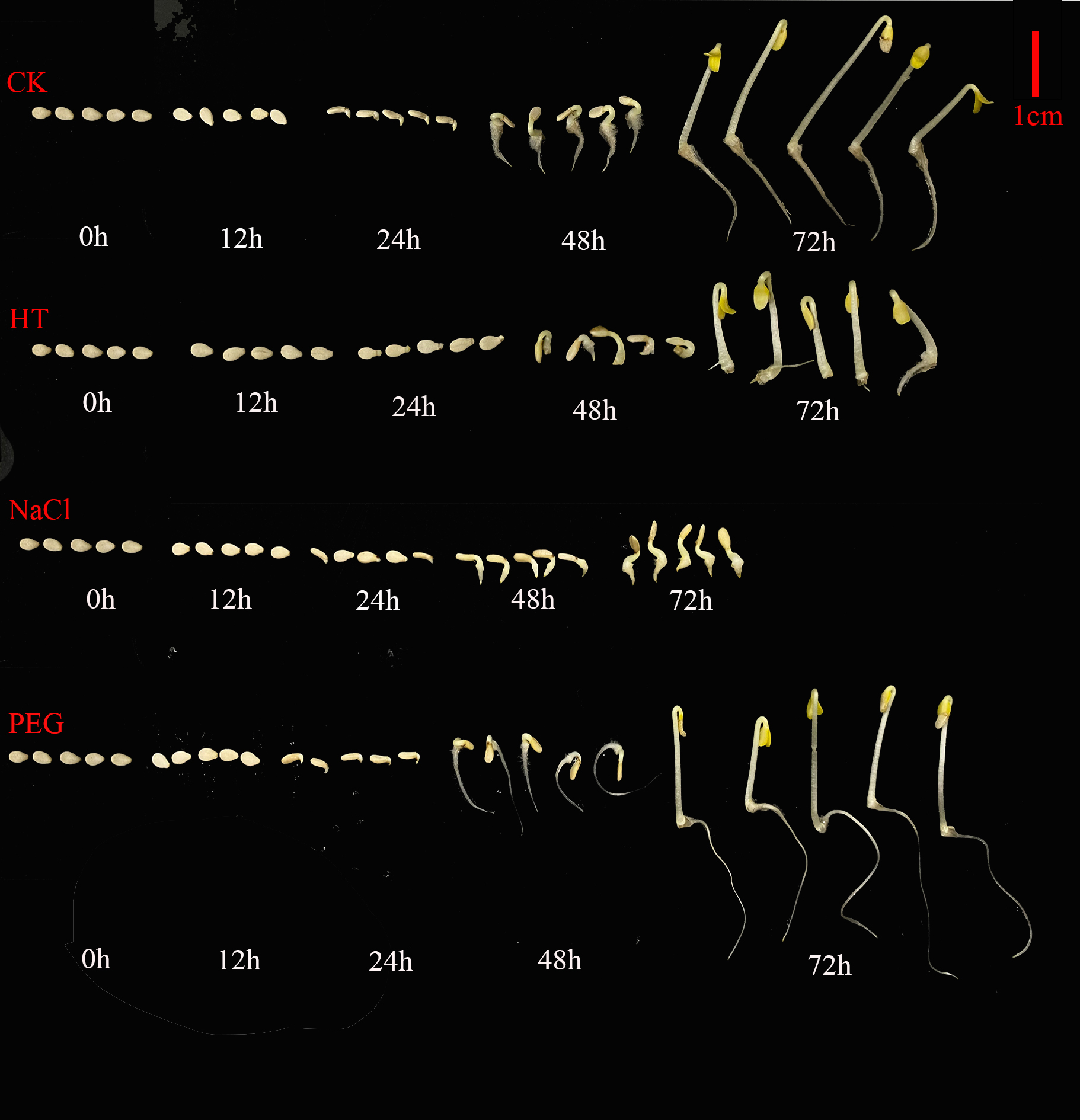

Supplement: Supplementary file 1 [file ijms-27-05470-s001.zip › Figure S2. Representative images of seed germination under abiotic stresses.jpg]
